# Immunohistochemical Inflammation in Histologically Normal Appendices in Patients with Right Iliac Fossa Pain

**DOI:** 10.1007/s00268-021-06288-w

**Published:** 2021-08-15

**Authors:** Emmanouil Psaltis, Abed M. Zaitoun, Keith R. Neal, Dileep N. Lobo

**Affiliations:** 1 Gastrointestinal Surgery, Nottingham Digestive Diseases Centre, National Institute for Health Research (NIHR) Nottingham Biomedical Research Centre, Nottingham University Hospitals NHS Trust and University of Nottingham, Queen’s Medical Centre, Nottingham, UK; 2 Pathology, Nottingham Digestive Diseases Centre, National Institute for Health Research (NIHR) Nottingham Biomedical Research Centre, Nottingham University Hospitals NHS Trust and University of Nottingham, Queen’s Medical Centre, Nottingham, UK; 3 Public Health and Epidemiology, Nottingham Digestive Diseases Centre, National Institute for Health Research (NIHR) Nottingham Biomedical Research Centre, Nottingham University Hospitals NHS Trust and University of Nottingham, Queen’s Medical Centre, Nottingham, UK; 4 MRC Versus Arthritis Centre for Musculoskeletal Ageing Research, School of Life Sciences, University of Nottingham, Queen’s Medical Centre, Nottingham, UK

## Abstract

**Background:**

Histologically normal appendices resected for right iliac fossa pain in children demonstrate immunohistochemical markers of inflammation. We aimed to establish if subclinical inflammation was present in histologically normal appendices resected from adults with right iliac fossa pain.

**Methods:**

Immunohistochemistry was performed on formalin-fixed paraffin-embedded appendices for tumour necrosis factor (TNF)-α, interleukin (IL)-6, IL-2R and serotonin in four groups: Group I (*n* = 120): uncomplicated appendicitis, Group II (*n* = 118): complicated appendicitis (perforation or gangrene), Group III (*n* = 104): histologically normal appendices resected for right iliac fossa pain and Group IV (*n* = 106) appendices resected at elective colectomy. Expression was quantified using the H-scoring system.

**Results:**

Median, interquartile range expression of TNF-α was increased in Groups I (5.9, 3.1–9.8), II (6.8, 3.6–12.1) and III (9.8, 6.2–15.2) when compared with Group IV (3.0, 1.4–4.7, *p* < 0.01). Epithelial expression of IL-6 in Groups II (44.0, 8.0–97.0) and III (71.0, 18.5–130.0) was increased when compared with Group IV (9.5, 1.0–60.2, *p* < 0.01). Expression of mucosal IL-2R in Groups I (47.4, 34.8–69.0), II (37.8, 25.4–60.4) and III (18.4, 10.1–34.7) was increased when compared with Group IV (2.8, 1.2–5.7, *p* < 0.01). Serotonin content in Groups I (3.0, 0–30.0) and II (0, 0–8.5) was decreased when compared with Groups III (49.7, 16.7–107.5) and IV (43.5, 9.5–115.8, *p* < 0.01).

**Conclusion:**

Histologically normal appendices resected from symptomatic patients exhibited increased proinflammatory cytokine expression on immunohistochemistry suggesting the presence of an inflammatory process not detected on conventional microscopy.

**Supplementary Information:**

The online version contains supplementary material available at 10.1007/s00268-021-06288-w.

## Introduction

Wang et al. [1] first demonstrated an activated immune system in children with histologically normal appendices, reporting that 25% of samples exhibited increased tumour necrosis factor-α (TNF-α) and interleukin (IL)-2 mRNA expression in germinal centres and the *lamina propria*. Nemeth et al. [2] subsequently showed that the expression of cyclo-oxygenase-2 in histologically normal appendices was similar to that in inflamed samples. Prostaglandin E_2_, inducible nitric oxide synthase and major histocompatibility complex Class II antigens were also strongly expressed in more than half of the histologically normal appendices [2]. Gene analysis has shown that expression of TNF-α, IL-12, interferon-γ, IL-4, IL-2 and IL-15 in samples of acute appendicitis differed when compared with controls [3]. However, the aforementioned studies were in children and had small sample sizes.

The aim of this study was to investigate the immunohistochemical expression of TNF-α, IL-6, IL-2R and serotonin in appendices resected from adult patients with symptoms of acute appendicitis, including those with histologically normal appendices. These findings were compared with a control group that included uninflamed appendices resected as part of an elective colectomy for cancer and were correlated with clinical features, laboratory investigations and intraoperative findings.

## Methods

Formalin-fixed paraffin-embedded appendix specimens were selected in a consecutive order in patients over the age of 16 years who underwent emergency appendicectomy between May 2011 and September 2013. The haematoxylin and eosin-stained histology slides were re-examined by a senior histopathologist (AMZ) blinded to the initial diagnosis. Samples were included when the initial diagnosis matched with that of the histopathologist who re-examined them. Samples with sub-serosal inflammation and healthy mucosa were excluded as this could indicate an inflammatory process arising elsewhere in the abdomen. The appendices for the control group were obtained from patients who underwent elective colectomy for cancer. The specimens were allocated to four groups (Table 1). The tissue architecture of the specimens from patients with right iliac fossa pain and histologically normal appendices and those undergoing colectomy (controls) was entirely normal (i.e. no features of acute or chronic inflammation) on conventional histology.

**Table 1:** Study groups and patient demographics

Groups	*N*	Sex		Age (years)
		Male	Female	Median (IQR)
Uncomplicated appendicitis (inflammation without gangrene or perforation)	120	65	54	30.0 (22.0–45.0)
Complicated appendicitis (inflammation with perforation or gangrene)	118	72	46	34.0 (21.0–49.2)
Normal (histologically normal appendices in patients with right iliac fossa pain)	104	44	60	22.0 (18.0–29.0)
Control (histologically normal appendices from elective colectomy)	106	46	57	70.5 (61.0–79.0)

5 μm consecutive sections were cut, de-paraffinised and heated to 98 °C to achieve epitope retrieval, and then blocked with peroxidase and protein. Subsequently, the samples were incubated with primary antibodies against TNF-α, IL-6, IL-2R and serotonin (Table 2). The Novolink™ polymer detection system Leica® RE7280-K with horseradish peroxidase linker antibody conjugates and 3,3′-diaminobenzidine chromogen was applied for enzyme–substrate labelling. Each substrate was applied for 5 min and counterstained with haematoxylin for 6 min. Finally, the slides were mounted in non-aqueous dibutyl phthalate with xylene mounting medium.

**Table 2:** Primary antibodies used in this study and details on their optimal immunostaining conditions

Cellular marker	Primary antibody	Clone	Cellular location	Antigen retrieval	Concentration/dilution	Incubation time	Positive control tissue	Supplier (product No.)
TNF-α	Anti-TNF-α mouse monoclonal	2C8	Cytoplasm	HIER with citrate buffer	7.5 μl/ml	1 h at RT	Inflamed colon	LSBio, Cambridge, UK (LS-B7268)
IL-6	Anti-IL-6 mouse monoclonal	10C10	Cytoplasm	HIER with citrate buffer	1:50	1 h at RT	Normal colon	Leica, Cambridge, UK (NCL-L-IL6)
IL-2R	Anti-IL-2R mouse monoclonal	4C9	Cell membrane	HIER with citrate buffer	1:400	Overnight at 4 °C	Tonsil	Leica, Cambridge, UK (NCL-CD25-305)
Serotonin	Anti-serotonin mouse monoclonal	5HT-H209	Cytoplasm	HIER with citrate buffer	1:100	30 min at RT	Carcinoid	Dako, Glostrup, Denmark (M0758)

*HIER* heat-induced epitope retrieval, *RT* room temperature

Manual immunohistochemistry assessment was chosen over computer-aided assessment as some of the inflammatory markers were detected in the membrane and some in the cytoplasm. The immunostaining evaluations were done in full sections by two observers (AMZ and EP). Ten randomly selected fields were scored for each specimen and their average formed the final score for each specimen. The expression of TNF-α and IL-2R was quantified by counting the positively stained cells [4]. The expression of IL-6 was semi-quantified using the H-scoring system [5]. The H-score was calculated by multiplying the percentage of positive cells (0–100) by a number representing the intensity of immune-reactivity (1 for weak, 2 for moderate and 3 for strong), giving a maximum score of 300. Serotonin content was semi-quantified using a modified H-scoring system due to the small number of positive cells. Instead of using the percentage of the stained cells, the number of positive cells was counted and multiplied by a number representing the intensity of immunoreactivity (1 for weak, 2 for moderate and 3 for strong).

Clinical data, as documented on the day of admission, were also collected. The duration of symptoms, severity of pain (on a scale of 1–10), previous episodes of right iliac fossa pain, presence of nausea/vomiting or localised peritonism, first and highest preoperative body temperature as well as the length of pre- and post-operative stay were recorded. The presence of a faecolith was assessed on imaging results, histopathology report and the operative record. Regarding the histologically normal appendices, the operative records were reviewed, and the appearance of the appendix was recorded. Appendices described as injected, engorged or hyper-vascular were classified as inflamed on operative description. White cell count (WCC), neutrophil count, lymphocyte count, neutrophil-to-lymphocyte ratio (NLR) and C-reactive protein (CRP) concentrations at admission were recorded.

### Ethics and consent

The study protocol was approved by the Health Research Authority (18/HRA/0292), and the need to obtain informed consent was waived.

### Statistical analysis

Statistical analysis was performed on IBM® SPSS® statistics software v24 (IBM Corp., Armonk, NY, USA). Data were expressed as *n* (%) or median, interquartile range (IQR). The Mann–Whitney U test was used to compare two groups and the Kruskal–Wallis H test to compare three or more groups. Cross-tabulation and the Chi-square test were used for categorical variables. Differences were considered statistically significant at *p* < 0.05. The Bonferroni correction was applied when multiple comparisons were performed.

## Results

A total of 448 patients, 342 of whom had a clinical diagnosis of acute appendicitis, were studied (Table 1). Immunohistochemistry findings, and laboratory and clinical data are summarised in Table 3.

**Table 3:** Summary of immunohistochemistry and clinical findings

Variables	Patient groups				*p* value
	Uncomplicated acute appendicitis—inflammation without perforation or gangrene^a^	Complicated acute appendicitis—inflammation with perforation or gangrene^b^	Histologically normal appendices in patients with right iliac fossa pain^c^	Normal appendices resected as part of an elective colectomy (control group)^d^	
TNF-α	5.9 (3.2–9.9)	6.8 (3.6–12.1)	9.8 (6.2–15.2)	3.0 (1.4–4.7)	< 0.001* *a* versus *b* = 0.17, *a* versus *c* < **0.001**, *a* versus *d* < **0.001**, *b* versus *c* = **0.004**, *b* versus *d* < **0.001**, *c* versus *d* < **0.001**
IL-6					
Epithelial cells	9.0 (1.0–53.5)	44.0 (8.0–97.0)	71.0 (18.5–130.0)	9.5 (1.0–60.2)	< 0.001* *a* versus *b* < **0.001**, *a* versus *c* < **0.001**, *a* versus *d* = 0.89, *b* versus *c* = **0.04**, *b* versus *d* < **0.001**, *c* versus *d* < **0.001**
Inflammatory cells	8.0 (3.0–17.5)	22.5 (10.6–46.0)	21.0 (10.0–42.2)	8.3 (4.4–23.6)	< 0.001* *a* versus *b* < **0.001**, *a* versus *c* < **0.001**, *a* versus *d* = 0.07, *b* versus *c* = 0.63, *b* versus *d* < **0.001**, *c* versus *d* < **0.001**
IL-2R					
Mucosa	47.4 (34.8–69.0)	37.8 (25.4–60.4)	27.0 (20.2–42.4)	15.4 (7.9–24.8)	< 0.001* *a* versus b = **0.001**, *a *versus c < **0.001**, *a* versus d < **0.001**, *b* versus c < **0.001**, *b* versus d < **0.001**, *c* versus *d* < **0.001**
Submucosa	67.8 (50.5–87.6)	47.9 (30.9–69.4)	18.4 (10.1–34.7)	2.8 (1.2–5.7)	< 0.001* *a* versus *b* < **0.001**, *a* versus *c* < **0.001**, *a* versus *d* < **0.001**, *b* versus *c* < **0.001**, *b* versus *d* < **0.001**, *c* versus *d* < **0.001**
Serotonin					
Enterochromaffin cells	3.0 (0–30.0)	0 (0–8.5)	49.7 (16.7–107.5)	43.5 (9.5–115.8)	< 0.001* *a* versus *b* = **0.001**, *a* versus *c* < **0.001**, *a* versus *d* < **0.001**, *b* versus *c* < **0.001**, *b* versus *d* < **0.001**, *c* versus *d* = 0.60
Subepithelial neuroendocrine cells	0 (0–2.0)	0 (0–0)	1.0 (0–5.7)	0 (0–3.0)	< 0.001* *a* versus *b* = **0.004**, *a* versus *c* = **0.001**, *a* versus *d* = 0.39, *b* versus *c* < **0.001**, *b* versus *d* < **0.001**, *c* versus *d* = 0.15
White cell count (× 10^9^/l)	13.6 (11.4–16.0)	15.2 (12.4–18.5)	10.0 (7.3–12.6)	8.3 (6.8–10.9)	< 0.001* *a* versus *b* = **0.007**, *a* versus *c* < **0.001**, *a* versus *d* < **0.001**, *b* versus *c* < **0.001**, *b* versus *d* < **0.001**, *c* versus *d* = **0.02**
Neutrophil count (× 10^9^/l)	11.0 (8.6–13.2)	12.6 (10.1–15.5)	6.2 (3.9–8.1)	5.7 (4.2–9.3)	< 0.001* *a* versus *b* = **0.002**, *a* versus *c* < **0.001**, *a* versus *d* < **0.001**, *b* versus *c* < **0.001**, *b* versus *d* < **0.001**, *c* versus *d* = 0.50
Lymphocyte count (× 10^9^/l)	1.6 (1.2–2.0)	1.2 (0.9–1.8)	2.1 (1.3–2.8)	1.5 (1.1–1.9)	< 0.001* *a* versus *b* = **0.002**, *a* versus *c* < **0.001**, *a* versus *d* = 0.44, *b* versus *c* < **0.001**, *b* versus *d* = **0.01**, *c* versus *d* < **0.001**
Neutrophil-to-lymphocyte ratio (NLR)	7.1 (4.6–10.6)	9.5 (6.5–15.2)	2.8 (1.6–6.0)	3.7 (2.3–7.1)	< 0.001* *a* versus *b* < **0.001**, *a* versus *c* < **0.001**, *a* versus *d* < **0.001**, *b* versus *c* < **0.001**, *b* versus *d* < **0.001**, *c* versus *d* = **0.02**
C-reactive protein (mg/l)	39.0 (10.0–90.0)	77.0 (24.0–170.0)	5.0 (5.0–23.7)	18.0 (5.0–71.0)	< 0.001* *a* versus *b* = **0.001**, *a* versus *c* < **0.001**, *a* versus *d* = 0.10, *b* versus *c* < **0.001**, *b* versus *d* = **0.001**, *c* versus *d* = **0.004**
Duration of pain (days)	2.0 (1.0–2.0)	2.0 (1.0–3.0)	2.0 (1.0–3.0)	N/A†	0.62* *a* versus *b* = 0.87, *a* versus *c* = 0.11, *b* versus *c* = 0.18
Severity of pain	8.0 (4.2–8.7)	8.0 (7.0–10.0)	8.0 (5.0–9.0)	N/A†	0.04* *a* versus *b* = **0.01**, *a* versus *c* = 0.68, *b* versus *c* = 0.07
Temperature (°C)					
First	37.2 (36.8–37.7)	37.4 (37.0–38.0)	37.1 (36.6–37.5)	N/A†	0.005* *a* versus *b* = **0.04**, *a* versus *c* = 0.16, *b* versus *c* = **0.002**
Highest	37.6 (37.2–38.1)	38.0 (37.5–38.7)	37.4 (37.1–37.8)	N/A†	< 0.001* *a* versus *b* < **0.001**, *a* versus *c* = **0.02**, *b* versus *c* < **0.001**
Hospital stay (days)					
Preoperative	1.0 (0–1.0)	1.0 (0–1.0)	1.0 (1.0–2.0)	N/A†	0.01* *a* versus *b* = 0.79, *a* versus *c* = **0.006**, *b* versus *c* = **0.01**
Post-operative	1.0 (1.0–3.0)	2.0 (1.0–5.0)	1.0 (1.0–2.0)	N/A†	< 0.001* *a* versus *b* = **0.004**, *a* versus *c* = 0.06, *b* versus *c* < **0.001**
Nausea/vomiting [*n* (%)]	78 (71%)	81 (75%)	63 (66%)	N/A†	0.39* *a* versus *b* = 0.49, *a* versus *c* = 0.47, *b* versus *c* = 0.17
Previous episodes of RIF pain [*n* (%)]	16 (14%)	4 (4%)	22 (23%)	N/A†	< 0.001* *a* versus *b* = **0.006**, *a* versus *c* = 0.10, *b* versus *c *< **0.001**
Localised peritonism [*n* (%)]	81 (73%)	79 (73%)	53 (56%)	N/A†	0.01* *a* versus *b* = 0.97, *a* versus c = **0.01**, *b* versus *c* = **0.01**
Faecolith [*n* (%)]	12 (10%)	30 (25%)	27 (26%)	N/A†	0.003* *a* versus b = **0.02**, *a* versus c = **0.002**, *b* versus *c* = 0.92

Data are presented as median (interquartile range) or *n* (%)

*Statistical significance for differences between 3 or more groups was calculated using the Kruskal–Wallis *H* test. Cross-tabulation and the Chi-square test were used for categorical variables. Significance for differences between two groups (e.g. *a* versus *b*) calculated using the Mann–Whitney *U* test. Statistically significant differences are shown in bold font

^†^Data on the clinical signs and symptoms to suggest appendicitis were not collected for the control group

**Fig. 1:**
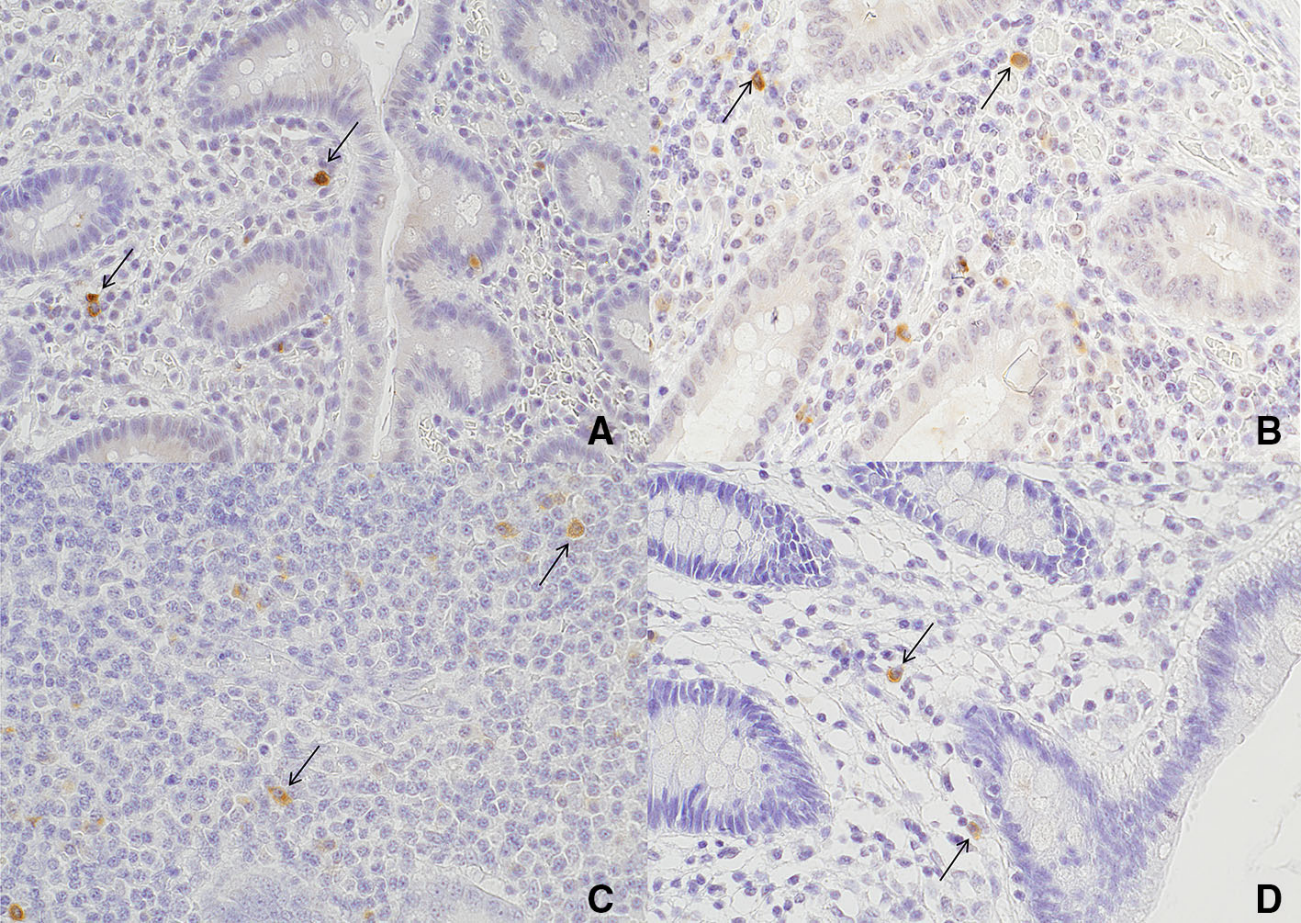
Expression of TNF-α (brown cytoplasmic staining) in monocytes (black arrows) using immunohistochemical staining at × 400 magnification. Expression of TNF-α in monocytes of patients with uncomplicated acute appendicitis (**a**); complicated acute appendicitis (**b**); histologically normal appendices (**c**); and patients from the control group (**d**). The vast majority of monocytes that expressed TNF-α were macrophages

**Fig. 2:**
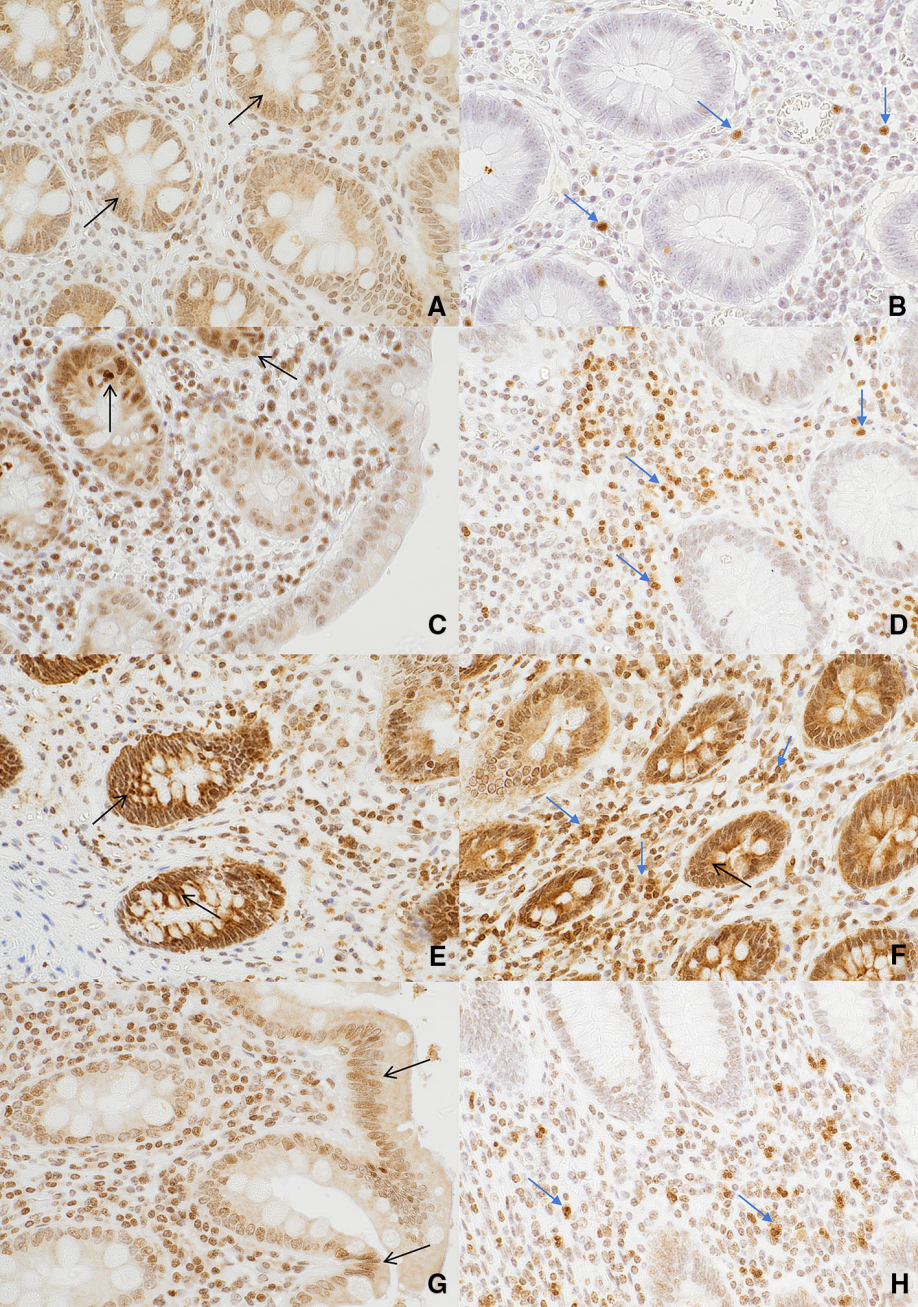
Expression of IL-6 (brown cytoplasmic staining) in epithelial (black arrows) and inflammatory (blue arrows) cells using immunohistochemical staining at × 400 magnification. Expression of IL-6 in epithelial (**a**) and inflammatory (**b**) cells in patients with uncomplicated acute appendicitis; epithelial (**c**) and inflammatory (**d**) cells in patients with complicated acute appendicitis; epithelial (**e**) and inflammatory (**f**) cells in patients with histologically normal appendices; and epithelial (**g**) and inflammatory (**h**) cells in patients from the control group. IL-6 expressed by both mononuclear and polymorphonuclear cells

Anti-TNF-α antibody staining was demonstrated in monocytes in the mucosa (Fig. 1), with most being macrophages. Immunohistochemical staining with anti-IL-6 antibody revealed cytoplasmic staining in the epithelial and inflammatory cells of the appendiceal mucosa (Fig. 2), with staining being observed in both mononuclear and polymorphonuclear cells.

**Fig. 3:**
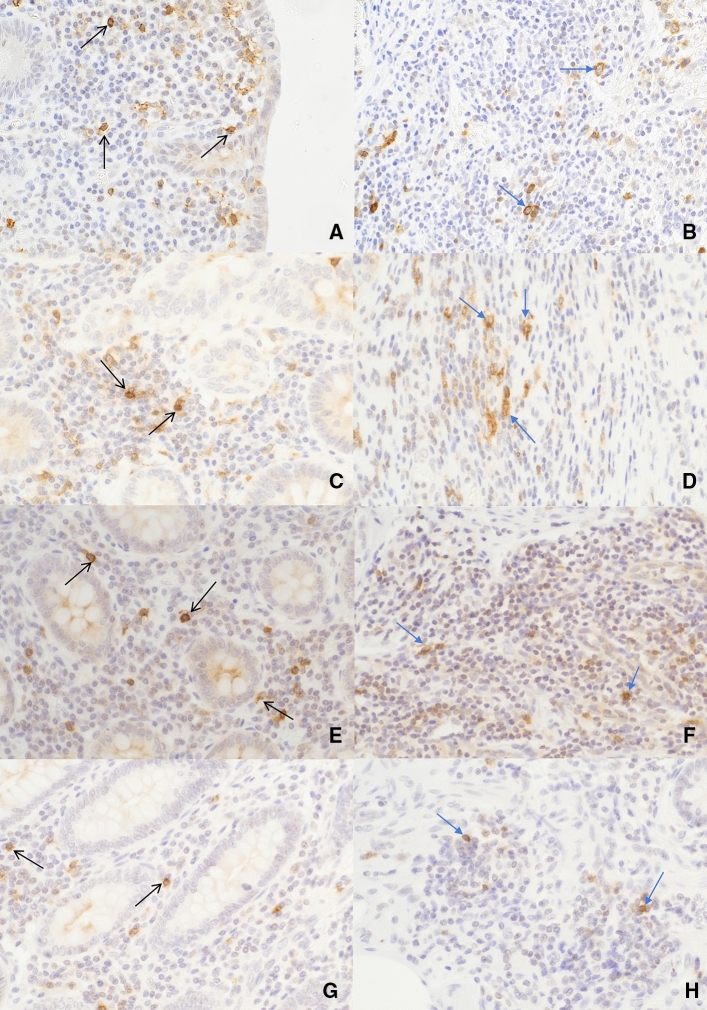
Expression of IL-2R (brown membrane staining) in the mucosa (black arrows) and submucosa (blue arrows) using immunohistochemical staining at × 400 magnification. Expression of IL-2R in the mucosa (**a**) and submucosa (**b**) of patients with uncomplicated acute appendicitis; mucosa (**c**) and submucosa (**d**) of patients with complicated acute appendicitis; mucosa (**e**) and submucosa (**f**) of patients with histologically normal appendices; and mucosa (**g**) and submucosa (**h**) of patients from the control group. Il-2R was expressed by lymphocytes

Immunohistochemical staining with anti-IL-2R antibody revealed membrane staining in the lymphocytes of the appendiceal mucosa and submucosa (Fig. 3). The germinal centres exhibited extremely intense IL-2R expression making it impossible to quantify. Therefore, submucosal IL-2R expression was quantified between the germinal centres.

**Fig. 4:**
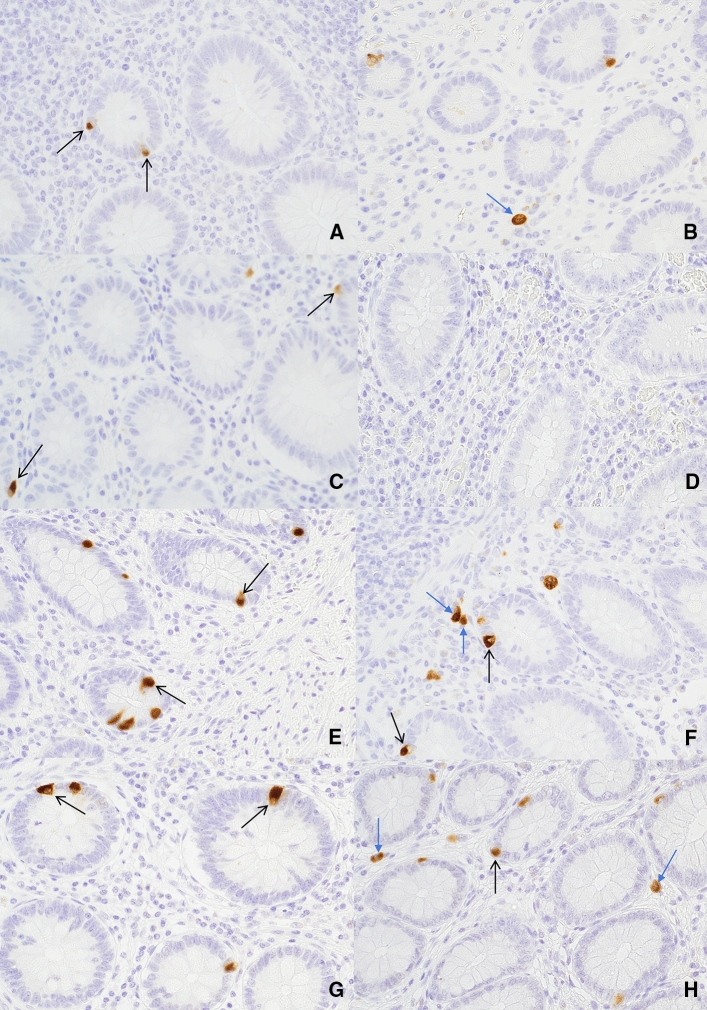
Serotonin contents (brown cytoplasmic staining) of enterochromaffin (ECC) and subepithelial neuroendocrine cells (SNC) using immunohistochemical staining at × 400 magnification. Serotonin contents of ECCs (**a**) and SNCs (**b**) in patients with uncomplicated acute appendicitis; ECCs (**c**) and SNCs (**d**) in patients with complicated acute appendicitis; ECCs (**e**) and SNCs (**f**) in patients with histologically normal appendices; and ECCs (**g**) and SNCs (**h**) in patients from the control group. ECCs (black arrows) appeared as small polygonal cells located in the crypts between the intestinal villi. SNCs (blue arrows) appeared as solitary or small clusters close to the crypts

Immunohistochemistry with anti-serotonin antibody demonstrated cytoplasmic staining in the enterochromaffin cells (ECC) within the crypts of the epithelium and subepithelial neuroendocrine cells (SNC) in the *lamina propria* (Fig. 4). SNCs appeared as either solitary cells or small clusters close to the crypts.

There were no significant differences in the duration of symptoms or severity of pain when patients with histologically normal appendices were compared with those with uncomplicated or complicated appendicitis (Table 3). However, those with uncomplicated appendicitis had significantly less severity of pain when compared with those with complicated appendicitis. There was no significant difference in the presence of nausea and vomiting across the three groups (excluding the control group).

The operating surgeon described the appendix as inflamed in 51% of the patients with right iliac fossa pain and histologically normal appendices. However, the expression of the studied inflammatory markers and the clinical parameters did not differ between patients whose appendix appeared normal and those whose appendix appeared inflamed to the naked eye.

The expression of the inflammatory markers and clinical parameters in the presence of previous episodes of right iliac fossa pain, localised peritonism and faecolith for patients from the three study groups are summarised in Supplementary Tables 1–3, respectively. There were no statistically significant differences for any of these parameters within each group after applying the Bonferroni correction for multiple testing.

## Discussion

This is the first study to present experimental findings on the immunohistochemical expression of inflammatory markers combined with clinical data, laboratory investigations and intraoperative findings encompassing the entire spectrum of appendicitis in adults. It has shown similarities in immunohistochemical expression of inflammatory markers in histologically normal appendices resected for right iliac fossa pain and histologically inflamed appendices, changes not seen in the control group.

The distinctively different cytokine expression between patients with complicated and uncomplicated acute appendicitis could indicate that the inflammatory response in complicated acute appendicitis is either prolonged due to delay in treatment or more severe suggesting a different immunopathogenesis. As IL-6 is a potent procoagulant leading to gangrene and is associated with the severity of the inflammatory process, the findings of increased IL-6 expression in complicated acute appendicitis support this [6–10]. Serotonin is an effective vasoconstrictor that could also contribute to ischaemia [11], as evidenced by the observed difference in serotonin contents between patients with complicated and uncomplicated acute appendicitis. TNF-α is also a potent proinflammatory mediator that has been associated with complicated acute appendicitis as well as local ischaemia [12]. However, as the differences in TNF-α expression between complicated and uncomplicated acute appendicitis samples were not statistically significant it could be acting synergistically with other cytokines in the development of complicated acute appendicitis.

The clinical data also favoured the concept that complicated acute appendicitis is not just a progression of an inflammatory process. Although body temperature, WCC and CRP concentrations in patients with complicated acute appendicitis were higher, the duration of the symptoms was similar in the complicated and uncomplicated appendicitis groups.

The increased expression of TNF-α, IL-6 and IL-2R in the histologically normal appendices resected for right iliac fossa pain could indicate an activated immune system as all three markers are essential for the acute inflammatory response [3, 13–15]. Abnormal growth of intestinal bacteria damages the epithelial barrier allowing increased bacterial translocation, which can trigger an innate immune response through communication with antigen presenting cells (APCs) such as macrophages and dendritic cells [16]. Viruses such as the adenovirus and rotavirus have also been implicated in the pathogenesis of acute appendicitis, especially in children and the type and intensity of inflammation may be related to the pathogen involved [17]. Activated macrophages produce proinflammatory mediators and upregulate the expression of cytokines such as TNF-α and IL-6, perpetuating intestinal inflammation [18, 19]. APCs also present antigens to B and T lymphocytes leading to activation of the adaptive immunity [16]. This is followed by rapid synthesis of IL-2/IL-2R [16, 20] which further promotes T and B lymphocyte proliferation [21]. Therefore, IL-2 acts synergistically with other cytokines for an optimal immune response.

In patients with acute appendicitis, the upregulated cytokines in the peritoneal fluid were both pro- and anti-inflammatory, whereas the plasma (systemic) cytokine profile was primarily anti-inflammatory [8]. IL-6 has anti-inflammatory properties as it can restrict recruitment of neutrophils by promoting their replacement by monocytes steering the inflammation towards chronicity [12, 14]. TNF-α also has some anti-inflammatory properties as it promotes the release of intestinal glucocorticoids which in turn control the activation of intestinal T lymphocytes [13]. Moreover, TNF-α can induce apoptosis of immune and non-immune cells [22]. Although apoptosis of T lymphocytes helps to control intestinal inflammation, apoptosis of epithelial cells compromises the integrity of the intestinal barrier allowing constant activation of intestinal immune cells by luminal antigens [23]. IL-2 is also crucial in downregulating immune responses and contributes in immune homeostasis [24]. Therefore, increased IL-6, TNF-α and IL-2R expression in histologically normal appendices could indicate an anti-inflammatory profile aiming to suppress an either abnormally long or abnormally regulated inflammatory response [24, 25]. However, increased TNF-α could compromise the integrity of the intestinal mucosa and lead to a vicious cycle of repeated inflammation [23]. Thus, several attempts in resolving an inflammatory response could lead to a chronic inflammatory status.

Our clinical data appeared to support this theory. The presence of previous episodes of right iliac fossa pain in patients with histologically normal appendices was significantly increased compared with patients with inflamed appendices. However, neither the severity of the pain nor the duration of symptoms between patients with histologically normal appendices and inflamed samples varied significantly. This could suggest that histologically normal appendices were either subjected to a recurrent inflammatory stimulus or the inflammatory response never successfully treated the initial stimulus.

Appendicitis is traditionally regarded as a progressing inflammatory process that will eventually lead to gangrene and perforation if left untreated. However, the fundamental differences in the cytokine expression between complicated and uncomplicated appendicitis could suggest a different immunopathogenesis for these two these entities and not just disease progression. This is supported by previously published data on the epidemiology of complicated and uncomplicated appendicitis [26, 27], and also by the clinical data presented in the present study as there was no statistically significant difference in the duration of symptoms between patients with uncomplicated and complicated appendicitis. Uncomplicated appendicitis could also resolve with primary antibiotic therapy [28] or even spontaneously [27] or with treatment with a placebo [29].

This retrospective single-centre study has some limitations. The control group consisted of patients with underlying malignancy and their age was significantly different from that of the rest study population. However, it was not possible to obtain specimens from young adults who were entirely healthy as a right colectomy for cancer is rare in this age group. Nevertheless, in 12 normal appendices removed from paediatric patients undergoing elective fundoplication for gastroesophageal reflux in a previous study, these normal control specimens showed almost complete absence of TNF-a and IL-2 mRNA expression [1], suggesting that the older age group of our control group did not substantially affect the results. In addition, we applied the Bonferroni correction, but as inflammatory markers move in the same direction this potentially underestimates the statistical significance of our findings. The use of immunohistochemistry alone without gene expression could also be considered a limitation. However, it assesses the expression of protein which is the final product of gene expression. Better understanding of gene expression in the entire spectrum of appendicitis is crucial and more studies are needed to investigate the differences in gene expression between different types of appendicitis and to understand the genetic predisposition to acute appendicitis and its pathogenesis [30].

Nevertheless, this study has shown that histologically "normal" appendices resected for right iliac fossa pain exhibited elevated cytokine expression at a stage where inflammation was undetectable on conventional histology. Whether this could progress to overt acute inflammation or to chronic symptoms if left untreated is yet unresolved.

## Supplementary Information

Below is the link to the electronic supplementary material.

Supplementary file1 (DOCX 31 KB)
