# Supplementary material for: Immunohistochemical Inflammation in Histologically Normal Appendices in Patients with Right Iliac Fossa Pain
Source: World J Surg. 2021 Aug 15;45(12):3592–602. doi: 10.1007/s00268-021-06288-w (PMC8572837; doi:10.1007/s00268-021-06288-w)
Supplement: Supplementary file 1 — Supplementary file1 (DOCX 31 KB) [file 268_2021_6288_MOESM1_ESM.docx]

**Supplementary Data**

**Supplementary Table 1:** Summary of the differences in the expression of the studied inflammatory markers and the levels of clinical parameters in patients with uncomplicated acute appendicitis due to the presence of previous episodes of RIF pain, localised peritonism and faecolith. The presence of faecolith and previous episodes of right iliac fossa pain was assessed on 317 patients as data was not available on 25 patients. The sign of peritonism was assessed on 314 patients as data was not available on 28 patients.

| **Variables** | | **Uncomplicated acute appendicitis** | | | | | | | | |
| --- | --- | --- | --- | --- | --- | --- | --- | --- | --- | --- |
|  |  | **Previous RIF pain**  (n=112) | | | **Localised peritonism**  (n=111) | | | **Faecolith**  (n=112) | | |
|  |  | **Present**  (n=16, 14%) | **Absent**  (n=96, 86%) | **p value** | **Present**  (n=81, 73%) | **Absent**  (n=30, 27%) | **p value** | **Present**  (n=12, 10%) | **Absent**  (n=100, 90%) | **p value** |
| **TNF-α** | | 6.0 (2.6-10.4) | 5.9 (3.4-9.6) | 0.86 | 6.0 (3.1-9.9) | 5.9 (3.4-8.8) | 0.78 | 6.8 (3.6-12.2) | 5.9 (3.3-9.6) | 0.46 |
| **IL-6** | **Epithelial** | 19.0 (1.5-63.7) | 8.0 (1.1-52.0) | 0.63 | 9.0 (1.0-71.5) | 7.0 (3.0-51.0) | 0.82 | 31.5 (731-123.0) | 8.0 (1.0-52.0) | 0.13 |
|  | **Inflammatory** | 4.8 (1.9-16.6) | 8.3 (3.4-17.7) | 0.37 | 6.8 (3.0-16.7) | 11.6 (1.8-19.0) | 0.74 | 8.0 (1.8-26.9) | 7.3 (3.0-17.0) | 0.85 |
| **IL-2R** | **Mucosa** | 44.2 (25.1-84.2) | 45.7 (36.0-68.2) | 0.60 | 47.4 (38.3-67.4) | 40.8 (30.8-70.6) | 0.26 | 54.5 (32.2-86.5) | 45.8 (35.2-68.2) | 0.68 |
|  | **Submucosa** | 61.4 47.5-81.1) | 67.8 (50.5-88.5) | 0.63 | 67.8 (50.2-89.5) | 65.7 (51.4-79.9) | 0.60 | 68.4 (41.1-89.5) | 67.9 (52.6-87.9) | 0.78 |
| **Serotonin** | **Enterochromaffin cells** | 6.5 (0-45.3) | 3.0 (0-27.0) | 0.46 | 4.0 (0-36.0) | 2.0 (0-9.0) | 0.15 | 1.0 (0-29.5) | 3.0 (0-30.0) | 0.57 |
|  | **Subepithelial neuroendocrine cells** | 0 (0-4.6) | 0 (0-2.0) | 0.49 | 0 (0-2.5) | 0 (0-2.0) | 0.54 | 0 (0-1.5) | 0 (0-2.2) | 0.36 |
| **White cell count (×10^9^/l)** | | 12.5 (11.0-14.9) | 13.7 (11.5-16.2) | 0.26 | 13.6 (11.4-16.3) | 13.7 (12.2-15.2) | 0.99 | 13.6 (11.9-17.7) | 13.6 (11.2-16.0) | 0.72 |
| **Neutrophil:Lymphocyte ratio** | | 7.7 (3.2-10.6) | 7.1 (4.6-10.7) | 0.66 | 7.4 (4.5-10.7) | 6.1 (4.7-9.3) | 0.29 | 7.7 (4.8-12.4) | 7.1 (4.6-10.6) | 0.66 |
| **C-reactive protein (mg/l)** | | 14.5 (5.0-63.0) | 40.0 (19.5-95.0) | **0.03*** | 37.5 (16.0-88.5) | 33.0 (5.0-72.2) | 0.46 | 58.0 (13.5-120.2) | 39.0 (11.5-87.0) | 0.54 |
| **Severity of pain** | | 8.0 (6.0-10.0) | 8.0 (4.0-8.0) | 0.25 | 8.0 (5.0-9.0) | 7.0 (4.0-8.0) | 0.20 | 7.5 (4.0-10.0) | 8.0 (5.0-8.0) | 0.91 |
| **Duration of symptoms (days)** | | 2.0 (1.0-3.7) | 2.0 (1.0-2.0) | 0.08 | 2.0 (1.0-3.0) | 1.0 (1.0-2.0) | **0.02*** | 1.0 (1.0-3.5) | 2.0 (1.0-2.0) | 0.47 |
| **Temperature (°C)** | **First** | 37.4 (36.8-37.8) | 37.1 (36.7-37.6) | 0.38 | 37.3 (36.8-37.7) | 37.0 (36.7-37.6) | 0.13 | 37.3 (36.7-37.6) | 37.2 (36.8-37.7) | 0.97 |
|  | **Highest** | 37.8 (37.2-38.8) | 37.6 (37.2-38.0) | 0.24 | 37.6 (37.3-38.2) | 37.5 (37.2-37.9) | 0.25 | 37.7 (37.4-37.9) | 37.6(37.2-38.1) | 0.34 |

Median (interquartile range).

Statistically significance for differences between 2 groups calculated using the Mann-Whitney U test. Due to multiple statistical testing, a Bonferroni-adjusted p value (0.0035) was applied to control for Type I error. Therefore, the observed differences marked * were not statistically significant.

**Supplementary Table 2:** Summary of the differences in the expression of the studied inflammatory markers and the levels of clinical parameters in patients with complicated acute appendicitis due to the presence of previous episodes of RIF pain, localised peritonism and faecolith. The presence of faecolith and previous episodes of right iliac fossa pain was assessed on 317 patients as data was not available on 25 patients. The sign of peritonism was assessed on 314 patients as data was not available on 28 patients.

| **Variables** | | **Complicated acute appendicitis** | | | | | | | | |
| --- | --- | --- | --- | --- | --- | --- | --- | --- | --- | --- |
|  |  | **Previous RIF pain**  (n=109) | | | **Localised peritonism**  (n=108) | | | **Faecolith**  (n=109) | | |
|  |  | **Present**  (n=4, 4%) | **Absent**  (n=105, 96%) | **p value** | **Present**  (n=79, 73%) | **Absent**  (n=29, 27%) | **p value** | **Present**  (n=30, 25%) | **Absent**  (n=79, 75%) | **p value** |
| **TNF-α** | | 4.2 (4.1- 6.9) | 7.0 (3.5-11.8) | 0.57 | 7.5 (4.2-11.3) | 5.9 (3.4-13.6) | 0.99 | 7.0 (3.2-11.3) | 6.6 (3.7-12.5) | 0.39 |
| **IL-6** | **Epithelial** | 50.0 (45.0-135.0) | 51.0 (8.5-103.0) | 0.46 | 50.0 (8.7-111.2) | 61.0 (8.6-92.0) | 0.90 | 65.0 (20.0-135.0) | 27.5 (5.7-94.2) | 0.64 |
|  | **Inflammatory** | 19.3 (18.0-47.4) | 24.7 (10.0-49.5) | 0.70 | 24.0 (10.9-50.6) | 22.4 (8.6-51.6) | 0.84 | 31.1 (18.0-61.1) | 20.0 (9.5-39.6) | 0.91 |
| **IL-2R** | **Mucosa** | 46.2 (44.4-48.0) | 36.5 (25.3-60.4) | 0.49 | 38.4 (25.0-60.7) | 35.0 (29.9-44.5) | 0.52 | 35.6 (18.4-60.3) | 38.4 (26.9-58.4) | 0.51 |
|  | **Submucosa** | 58.7 (32.1-78.5) | 48.4 (30.3-68.5) | 0.66 | 46.6 (29.8-67.0) | 55.6 (34.0-77.9) | 0.23 | 52.4 (30.5-70.2) | 47.0 (31.1-69.5) | 0.52 |
| **Serotonin** | **Enterochromaffin cells** | 25.5 (0-45.2) | 0 (0-9.3) | 0.22 | 0 (0-12.0) | 0 (0-1.5) | 0.23 | 0 (0-8.7) | 0 (0-8.5) | 0.74 |
|  | **Subepithelial neuroendocrine cells** | 1.0 (0-4.5) | 0 (0-0) | 0.18 | 0 (0-0) | 0 (0-1.0) | 0.74 | 0 (0-0) | 0 (0-0) | 0.63 |
| **White cell count (×10^9^/l)** | | 15.2 (12.7-20.8) | 15.2 (12.3-18.5) | 0.74 | 15.1 (11.9-18.0) | 15.4 (12.6-19.1) | 0.39 | 14.9 (9.9-18.5) | 15.4 (13.1-1834) | 0.20 |
| **Neutrophil:Lymphocyte ratio** | | 6.3 (37-12.9) | 10.1 (6.5-15.5) | 0.22 | 10.6 (6.4-14.7) | 8.5 (6.2-17.9) | 0.54 | 9.7 (5.2-16.4) | 9.2 (6.4-14.7) | 0.79 |
| **C-reactive protein (mg/l)** | | N/A****** | N/A****** | N/A****** | 85 (21.0-195.0) | 73.0 (47.0-162.0) | 0.98 | 147.0 (72.0-229) | 65.5 (20.0-161.5) | **0.02*** |
| **Severity of pain** | | 4.2 (4.0-6.5) | 7.0 (4.0-8.0) | 0.14 | 8.0 (7.0-10.0) | 8.0 (7.0-9.0) | 0.42 | 8.0 (7.2-9.0) | 8.0 (7.0-10.0) | 0.90 |
| **Duration of symptoms (days)** | | 1.0 (1.0-1.0) | 1.0 (2.0-3.0) | **0.03*** | 2.0 (1.0-3.0) | 2.0 (1.0-2.0) | 0.14 | 2.0 (1.0-3.0) | 2.0 (1.0-2.0) | 0.19 |
| **Temperature (°C)** | **First** | 36.7 (36.2-37.1) | 37.4 (37.0-38.0) | **0.02*** | 37.4 (37.0-38.0) | 37.3 (37.0-37.7) | 0.61 | 37.4 (37.0-38.3) | 37.4 (37.0-37.9) | 0.43 |
|  | **Highest** | 37.6 (37.2-38.5) | 38.1 (37.5-38.7) | 0.41 | 38.1 (37.5-38.7) | 37.8 (37.4-38.6) | 0.30 | 38.5 (37.6-38.7) | 38.0 (37.5-38.6) | 0.21 |

Median (interquartile range).

Statistically significance for differences between 2 groups calculated using the Mann-Whitney U test. Due to multiple statistical testing, a Bonferroni-adjusted p value (0.0035) was applied to control for Type I error. Therefore, the observed differences marked * were not statistically significant. ****** several values of C-reactive protein concentrations were not available and statistical analysis could not be performed.

**Supplementary Table 3:** Summary of the differences in the expression of the studied inflammatory markers and the levels of clinical parameters in patients with histologically "normal" appendices due to the presence of previous episodes of RIF pain, localised peritonism, faecolith. The presence of faecolith and previous episodes of right iliac fossa pain was assessed on 317 patients as data was not available on 25 patients. The sign of peritonism was assessed on 314 patients as data was not available on 28 patients.

| **Variables** | | **Histologically "normal" appendices** | | | | | | | | |
| --- | --- | --- | --- | --- | --- | --- | --- | --- | --- | --- |
|  |  | **Previous RIF pain**  (n=96) | | | **Localised peritonism**  (n=95) | | | **Faecolith**  (n=96) | | |
|  |  | **Present**  (n=22, 23%) | **Absent**  (n=74, 77%) | **p value** | **Present**  (n=53, 56%) | **Absent**  (n=42, 44%) | **p value** | **Present**  (n=27, 26%) | **Absent**  (n=69, 74%) | **p value** |
| **TNF-α** | | 11.4 (7.0-16.1) | 9.6 (5.8-15.2) | 0.29 | 9.7 (6.8-15.3) | 9.7 (5.5-14.6) | 0.56 | 11.2 (6.7-16.1) | 9.7 (6.9-14.4) | 0.50 |
| **IL-6** | **Epithelial** | 69.0 (7.6-172.5) | 74.5 (24.0-130.5) | 0.88 | 81.0 (19.7-131.0) | 70.0 (20.2-142.5) | 0.97 | 26.4 (19.6-44.4) | 27.2 (20.4-39.9) | 0.87 |
|  | **Inflammatory** | 27.0 (6.1-52.7) | 20.5 (11.3-41.2) | 0.93 | 20.0 (10.2-40.1) | 27.0 (11.0-44.3) | 0.50 | 13.6 (7.0-27.0) | 21.6 (12.0-39.6) | 0.05 |
| **IL-2R** | **Mucosa** | 27.2 (22.3-43.2) | 28.3 (19.5-43.6) | 0.85 | 30.0 (22.8-45.5) | 24.3 (18.3-39.9) | 0.98 | 25.0 (13.2-96.0) | 53.5 (19.1-112.3) | 0.27 |
|  | **Submucosa** | 16.8 (7.6-31.8) | 21.6 (10.4-36.9) | 0.40 | 22.8 (10.2-37.7) | 17.0 (7.6-34.5) | 0.26 | 1.0 (0-3.0) | 2.0 (0-0.6) | 0.13 |
| **Serotonin** | **Enterochromaffin cells** | 33.3 (14.5-113.5) | 43.4 (16.3-95.5) | 0.96 | 33.6 (13.1-86.9) | 45.5 (1937-115.0) | 0.47 | 29.5 (11.0-87.0) | 82.5 (30.0-150.0) | **0.04*** |
|  | **Subepithelial neuroendocrine cells** | 2.0 (0-6.8) | 1.0 (0-4.0) | 0.36 | 1.0 (0-5.4) | 1.0 (0-5.2) | 0.87 | 20.0 (5.8-42.0) | 21.5 (11.5-43.4) | 0.33 |
| **White cell count (×10^9^/l)** | | 9.5 (6.3-10.5) | 10.2 (7.4-13.3) | 0.97 | 10.0 (7.5-12.6) | 10.1 (7.2-13.1) | 0.82 | 9.5 (7.3-11.3) | 10.1 (7.0-13.0) | 0.89 |
| **Neutrophil:Lymphocyte ratio** | | 1.9 (1.2-4.7) | 3.3 (1.7-6.3) | 0.73 | 2.9 (1.9-5.8) | 2.9 (1.4-8.6) | 0.90 | 3.3 (1.65-7.1) | 2.6 (1.5-5.8) | 0.40 |
| **C-reactive protein (mg/l)** | | 5.0 (5.0-8.7) | 7.0 (5.0-48.5) | **0.03*** | 5.0 (5.0-25.0) | 6.0 (5.0-43.7) | 0.64 | 5.0 (5.0-36.0) | 5.0 (5.0-24.0) | 0.98 |
| **Severity of pain** | | 8.0 (5.5-10.0) | 8.0 (5.0-9.0) | 0.51 | 8.0 (6.0-10.0) | 8.0 (4.0-8.5) | 0.20 | 8.0 (4.5-10.0) | 8.0 (5.0-9.0) | 0.72 |
| **Duration of symptoms (days)** | | 2.0 (1.0-3.5) | 2.0 (1.0-3.0) | 0.62 | 2.0 (1.0-3.0) | 2.0 (1.0-3.0) | 0.50 | 2.0 (1.0-3.0) | 2.0 (1.0-3.0) | 0.68 |
| **Temperature (°C)** | **First** | 37.1 (36.6-37.4) | 37.1 (36.6-37.6) | 0.84 | 37.1 (36.6-37.6) | 37.1 (36.5-37.5) | 0.85 | 37.1 (36.7-37.5) | 37.1 (36.5-37.5) | 0.76 |
|  | **Highest** | 37.2 (37.1-37.7) | 37.4 (37.1-37.9) | 0.37 | 37.3 (37.1-37.9) | 37.5 (37.1-37.8) | 0.60 | 37.3 (37.0-37.8) | 37.4 (37.1-37.8) | 0.42 |

Median (interquartile range).

Statistically significance for differences between 2 groups calculated using the Mann-Whitney U test. Due to multiple statistical testing, a Bonferroni-adjusted p value (0.0035) was applied to control for Type I error. Therefore, the observed differences marked * were not statistically significant.
